# Lanthanide complexes based on a conjugated pyridine carboxylate ligand: structures, luminescence and magnetic properties†

**DOI:** 10.1039/c9ra10975g

**Published:** 2020-02-10

**Authors:** Rong-fang Li, Rui-hao Li, Xin-fang Liu, Xin-hong Chang, Xun Feng

**Affiliations:** College of Chemistry and Chemical Engineering, Henan Key Laboratory of Function Oriented Porous Materials, Luoyang Normal University Luoyang Henan 471934 China fengx@lynu.edu.cn

## Abstract

Three lanthanide compounds have been synthesized, namely, {[Dy_2_(bpda)_3_(H_2_O)_3_]_4_·2H_2_O}(Dy-1), {[Sm(bpda)_2_·(H_2_O)]·H_2_O}_*n*_ (Sm-2) and {[Tb_2_(bpda)_3_(H_2_O)_3_]_4_·2H_2_O} (Tb-3) (H_2_bpda = 2,2′-bipyridine-6,6′-dicarboxylic acid). Their structures were determined by single crystal X-ray diffraction and characterized by elemental analysis, infrared spectroscopy and thermogravimetric analysis. Dy-1 and Tb-3 are isostructural with a conjugate bimolecular four-nuclear cluster structure constructed with intramolecular hydrogen bonds and they form a 3D supramolecular structure with intermolecular hydrogen bonding. Sm-2 is a one-dimensional chain structure and is further connected by intricate hydrogen bonds into a three-dimensional supramolecular structure. These three compounds exhibit significant characteristic luminescence from the ligand to the central Ln(iii) ion, which is found by solid-state photoluminescence measurement. Sm-2 exhibits a long luminescence lifetime and high fluorescence quantum yield. A slow relaxation phenomenon is observed for the dysprosium compound by measuring the alternating-current susceptibility at low temperature and the underlying mechanism was further confirmed by theoretical calculations.

## Abstract 2

The structures, fluorescence and magnetic properties of series three lanthanide complexes have been investigated. Dysprosium complex exhibit the single-molecule behavior.

## Graphical abstract

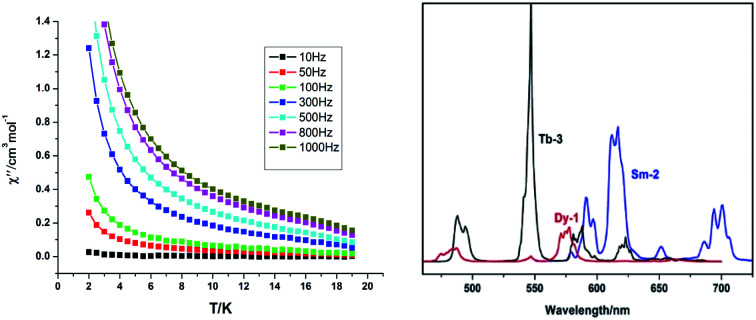

## 1. Introduction

Through the past decades, lanthanide compounds including europium(iii), terbium(iii) and samarium(iii) compounds have attracted much attention in the fields of biological analysis,^1^ magnetism,^2,3^ chemosensors,^4–6^ electroluminescent devices and laser systems.^7–11^ However, lanthanide(iii) ions usually have very low optical transition absorption coefficients, which greatly limits their practical applications. This disadvantage can be overcome by using highly absorbent ligands to efficiently sensitize lanthanide ions. After the introduction of suitable organic ligands, lanthanide compounds exhibit unique photophysical properties, such as strong luminescence, high quantum yield, long luminescence life and large Stokes shifts.^12,13^ Experiments show that H_2_bpda ligand is an efficient sensitizer for the lanthanide ions because the energy gaps between the ligand and lanthanum ions is suitable for the effective ligand-to-metal energy transfer. Moreover, H_2_bpda ligand can be coordinate to lanthanide ions in various modes, such as unidentate coordinating, bidentate chelating and bridging coordination since the carboxyl group of the ligand can be partially or completely dehydrogenated by adjusting the pH value.^14^ Thereby, the rich diversity of structures have the potential to have some fascinating properties. Meanwhile, the magnetic properties of lanthanide compounds are also an attractive field^15,16^ and they have provided an opportunity to shed light on tuning of the magnetic properties of Ln(iii) compound.^17^ Single-molecule magnets (SMM) exhibit magnetic bistability and quantum magnetic properties due to the existence of magnetic anisotropic energy barrier, which makes it a candidate material for ultra-high density information storage, quantum computing and molecular spintronic.^18^ The magnetic measurements in this article reveal that Dy-1 displays weak magnetic relaxation under a zero dc field. Combined with the *ab initio* calculations, the magnetic anisotropy and magnetic dynamic of Dy-1 were studied.

## 2. Experimental

### 2.1 Materials and physical measurements

H_2_bpda and other raw materials are analytical reagents, purchased from commercial channels, without further purification. Elemental analysis of carbon, hydrogen and nitrogen was performed on a Vario EL III elemental analyzer. Fourier-transform infrared (FT-IR) spectra (4000–400 cm^−1^) were collected in the solid state on an Avatar™ 360 E. S. P. IR spectrometer using KBr pellet. Using SDT 2960 thermogravimetric analyzer, the temperature rise rate is 10 °C min^−1^ (Al_2_O_3_ ceramic disc is the support) when nitrogen flow is 40 mL min^−1^ in the range of 30–800 °C, and thermogravimetric analysis (TGA) is carried out. Solid-state luminescence spectra, luminescence lifetimes and luminescence quantum yield (QY) of the three compounds were measured with an Edinburgh instrument FLS1000 fluorescence spectrometer at room temperature. Luminescence QY was also collected by the same Edinburgh FLS1000 which equipped with an integrating sphere. Magnetic susceptibility were measured with Quantum Design PPMS-XL9 VSM. DC variable-temperature magnetic susceptibilities were measured under a 0.1 T applied magnetic field in 2–300 K. The diamagnetic contribution calculated by Pascal constants was used to correct all the data.

### 2.2 Syntheses

H_2_bpda (0.036 g, 0.15 mmol), 0.5 mL Ln(NO_3_)_3_ (Ln = Dy, Sm, Tb) (0.1 mol L^−1^) and 12 mL deionized water were mixed together and stirred for several minutes, adjusting the pH value to 4 with hydrochloric acid. The mixture was placed in a 25 mL Teflon-lined autoclave, heated at 150 °C autogenic pressure for 3 days, and then cooled to room temperature at a rate of 1 °C h^−1^. After filtration, washing and drying, crystals of 1–3 were obtained suitable for X-ray diffraction analysis.

Dy-1: colorless block crystals. Yield: 0.032 g (58%) based on dysprosium element. Anal. calcd for C_144_H_98_Dy_8_N_24_O_61_ (%): C, 38.95; H, 2.22; N, 7.57. Found: C, 38.57; H, 2.26; N, 7.58. IR (cm^−1^): 3074–3380 br, 2351 m, 1630–1556 *vs.*, 1465 s, 1415 s, 1380 *vs.*, 1272 s, 1192 s, 1160 s 1087 m, 1018 s, 914 m, 856 s, 775 s, 678 s, 644 s, 570 s, 428 s.

Sm-2: yellow primrose blocks crystals. Yield: 0.0251 g (77%) based on samarium element. Anal. calcd for C_24_H_14_N_4_O_9_Sm (%): C, 44.16; H, 2.16; N, 8.58. Found: C, 44.17; H, 2.16; N, 8.59. IR (cm^−1^): 3075–3380 br, 2350 m, 1630–1554 *vs.*, 1465 s, 1415 s, 1380 *vs.*, 1271 s, 1190 s, 1160 s 1085 m, 1018 s, 915 m, 856 s, 775 s, 678 s, 645 s, 570 s, 428 s.

Tb-1: colorless block crystals. Yield: 0.021 g (75%) based on terbium element. Anal. calcd for C_144_H_98_N_24_O_61_Tb_8_ (%): C, 39.20; H, 2.24; N, 7.62. Found: C, 39.19; H, 2.25; N, 7.60. IR (cm^−1^): 3070–3460 br, 2353 m, 1630–1558 *vs.*, 1465 s, 1419 s, 1381 *vs.*, 1273 s, 1192 s, 1161 s, 1087 m, 1018 s, 914 m, 856 s, 775 s, 678 s, 644 s, 570 s, 428 s.

### 2.3 X-ray crystallography

The X-ray intensity data of compounds were collected on an Oxford Diffraction Super Nova area-detector diffractometer using mirror optics monochromatic MoKα radiation (*λ* = 0.71073 Å) at 296(8) K. Structures have been solved with olex2.solve and refined with ShelXL (2014) and olex2.refine.^19^ Crystal analytical data are shown in Table 1, and selected bond lengths and bond angles are shown in Table S1 of ESI.†

**Table 1:** Crystallographic data and structure refinements for 1 to 3^a^

Complexes	1	2	3
Empirical formula	C_144_H_98_N_24_O_61_Dy_8_	C_24_H_14_N_4_O_9_Sm	C_144_ H_98_N_24_O_61_Tb_8_
Formula weight	4440.46	652.77	4411.82
Crystal system	Monoclinic	Monoclinic	Monoclinic
Space group	*P*12/*c*_1_	*P*2(1)/*n*	*P*12/*c*_1_
Unit cell dimensions (Å, °)			
*a*	19.329(4)	6.780(2)	19.3615(3)
*b*	18.257(4)	27.939(8)	18.2756(2)
*c*	23.014(4)	11.546(4)	23.0963(4)
*α*	90.00	90.00	90
*β*	107.892(3)	101.630(4)	107.926(2)
*γ*	90.00	90.00	90
Volume (Å^3^), *Z*	7728(3)	2142.1(12)	7775.7(2)
Absorption coefficient (mm^−1^)	3.917	2.812	3.688
Calculated density (g cm^−3^)	1.908	2.0239	1.884
*F*(000)	4292.0	1281.0	4276.0
Final *R* indices [*I* > 2*σ*(*I*)]	*R*_1_ = 0.0495, w*R*_2_ = 0.1336	*R*_1_ = 0.0229, w*R*_2_ = 0.0603	*R*_1_ = 0.0279, w*R*_2_ = 0.0603
*R* indices (all data)	*R*_1_ = 0.0676, w*R*_2_ = 0.1502	*R*_1_ = 0.0255, w*R*_2_ = 0.066	*R*_1_ = 0.0349, w*R*_2_ = 0.0626
*R*_int_	0.0572	0.0211	0.0395
*T* (K)	293(2)	296(2)	296(2)
Largest difference in peak and hole (e Å^−3^)	1.415 and −2.785	0.7434 and −1.1433	0.765 and −1.022
Goodness-of-fit on *F*^2^	1.009	1.049	1.048

a *R* = ∑||*F*_0_| − |*F*_c_||/∑|*F*_0_|, w*R* = {∑[w(*F*_0_^2^ − *F*_c_^2^)^2^]/∑(*F*_0_^2^)^2^}^1/2^.

## 3. Results and discussion

### 3.1 Infrared spectroscopy and absorption spectra

**Fig. 1:**
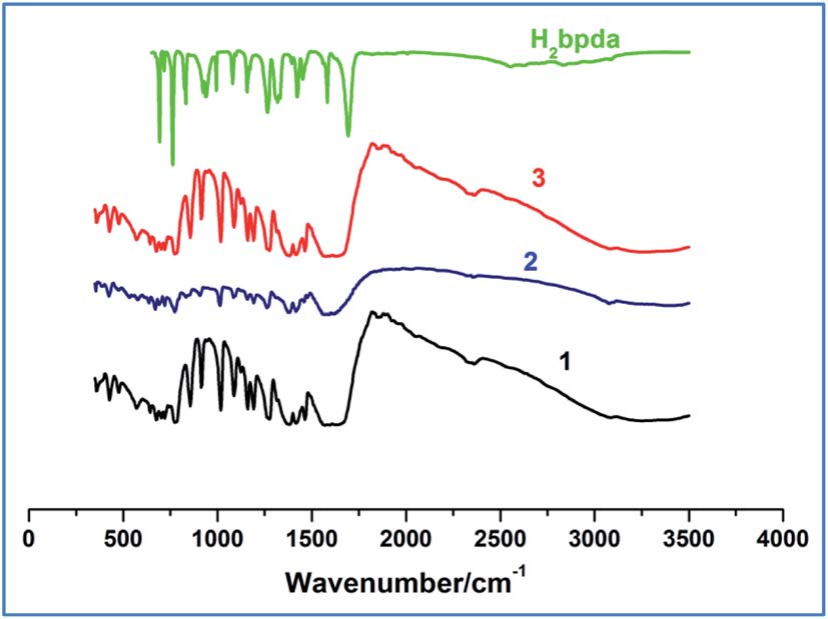
The IR spectra of H_2_bpda ligand and complex 1–3.

Within the range of 4000–400 cm^−1^, the IR spectra of 1–3 compounds and H_2_bpda were determined. As shown in Fig. 1, the similarity of the complexes 1 and 3 spectra suggested that they had similar coordination structures. The broad bands at 3070–3500 cm^−1^ are assigned to O–H stretching vibrations in Dy-1 and Tb-3, while this band is not obvious in Sm-2, indicating that there is hardly any hydroxyl group in this complex.

In the IR spectrum of H_2_bpda ligand, the bands at 1692 cm^−1^ and 1264–1325 cm^−1^ could be attributed to stretching vibration (*ν*(C=O)) and bending vibration (*δ*(O–H)) of carboxylic acid, respectively. These two bands disappeared in complexes 1–3 and two new ones of 1545–1667 cm^−1^ and 1378–1457 cm^−1^ appeared. The bands at 1545–1667 cm^−1^ could be attribute to the asymmetric stretching vibrations of carboxylate (*ν*_as_(COO^−^)), and the bands at 1378–1457 cm^−1^ could be ascribed to the symmetric stretching vibrations of carboxylate (*ν*_s_(COO^−^)) in the complexes.^20^ The separation (Δ*ν*) between *ν*_as_(COO^−^) and *ν*_s_(COO^−^) can be used to explain the coordination types of carboxyl groups in ligand. Therefore, the Δ*ν* values of 167–210 cm^−1^ in the spectra of compounds 1–3 suggest that the carboxylate groups may coordinate to the lanthanide ions *via* monodentate and bidentate coordination modes.^21,22^

**Fig. 2:**
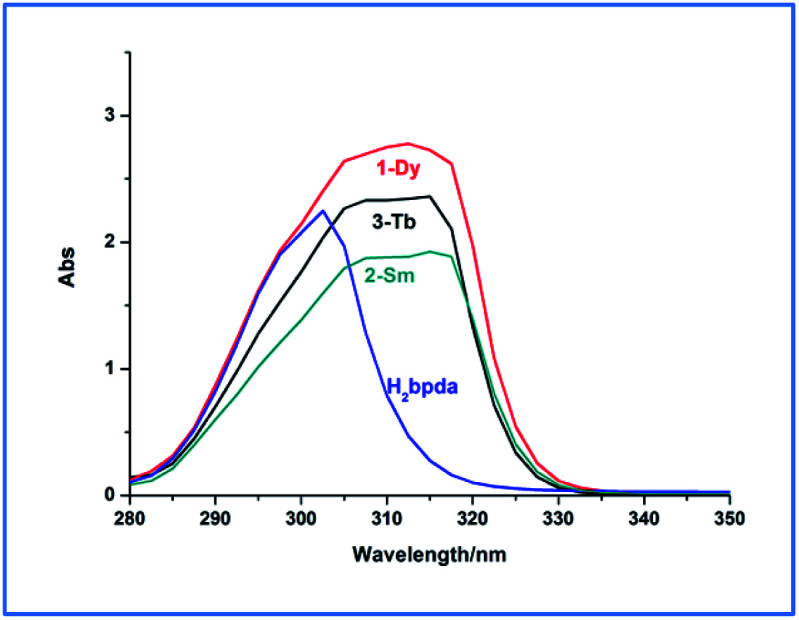
Electronic absorption spectra of the ligand and the complexes 1–3.

The UV-vis absorption spectra of the ligand and the three complexes were measured in DMF solvent. As shown in Fig. 2, the absorption peaks of H_2_bpda appeared at 302 nm, which could be ascribed to the n–π* or π–π* absorption of conjugate pyridine ring of the ligand. This peak disappears in the complexes and new absorption peaks appeared at 305–317 nm. Relative to the ligand, the peak red shift in the complexes indicated that the ligand coordinated to the lanthanide ions and lower the energy.^23^ In addition, compared with the ligand, the absorption spectra of lanthanide compounds changing, indicated that the coordination of the lanthanide ions significantly influence the energy levels of the ligands.^24^

### 3.2 Structural descriptions

**Fig. 3:**
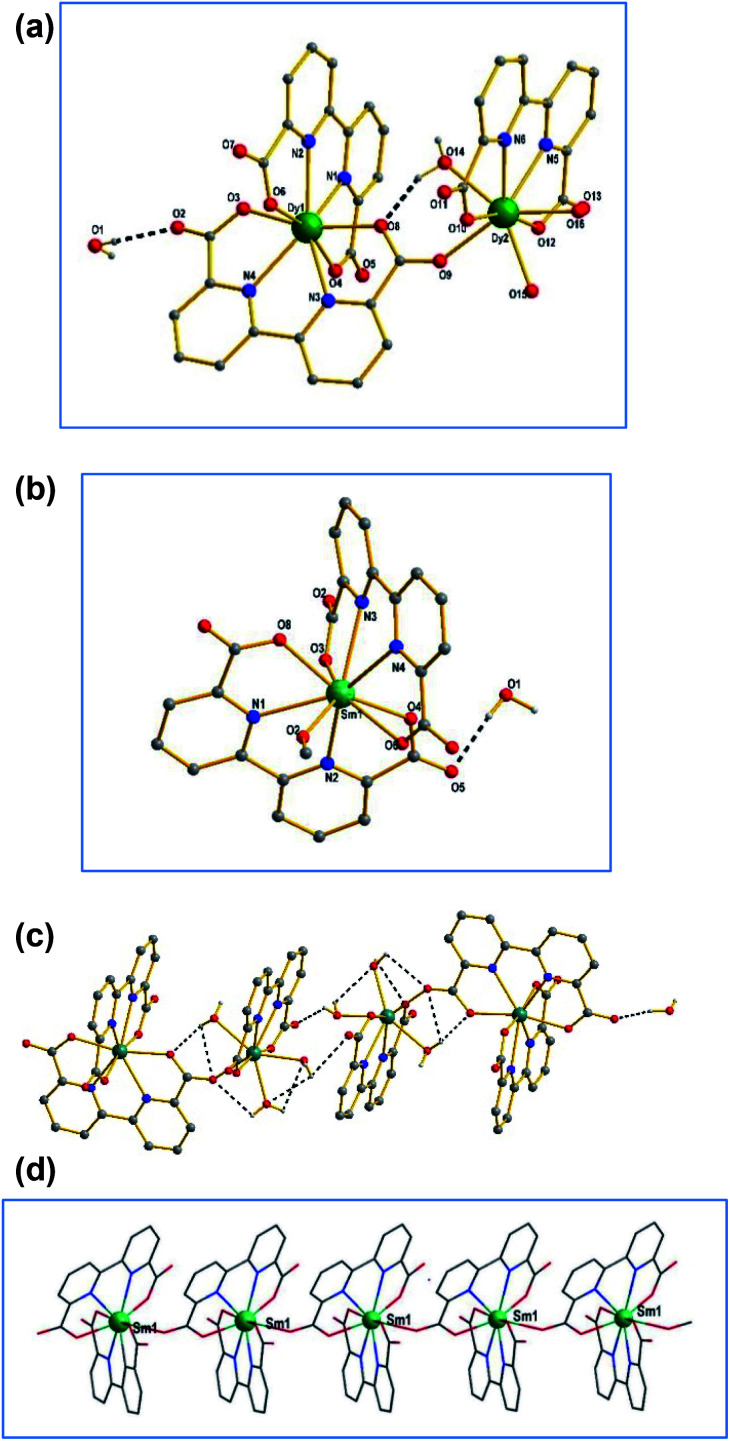
(a) The coordinated modes of Dy(iii) for compound 1. (b) The coordinated environment of Sm(iii) for Sm-2. (c) The pack structure connected by hydrogen bonds in Dy-1. (d) The one-dimensional chain structure of complex 2.

Complexes Dy-1 and Tb-3 are isostructural, hence only the structure of Dy-1 is discussed detailed as a representative. As shown in Fig. 3a, the structure unit of Dy-1 consists of two Dy(iii) ion, three bpda^2−^ ligand, three coordinated water molecules. Dy1 is eight-coordinated of which four oxygen atoms and four nitrogen atoms come from two bpda^2−^ ligands, respectively. Dy2 is also eight coordinated, where two oxygen atoms and two nitrogen atoms come from the same bpda^2−^ ligand and three oxygen atoms from three water molecules and the rest of the oxygen atom from another bpda^2−^ ligand. Dy–O bond distances are in the range of 2.277(2)–2.390(3) Å and Dy–N lengths in the range of 2.457(2)–2.501(3) Å, which indicate that oxygen atom has stronger coordination capacity than nitrogen atom. The adjacent dysprosium ions Dy1 and Dy2 are connected by O9 of the carboxyl group. The distance between Dy1 and Dy2 is 6.278 Å. The [Dy_2_(bpda)_3_(H_2_O)_3_] units are connected by hydrogen bonds between lattice water molecules and coordinated water molecules (Fig. 3c), forming a three-dimensional supramolecular structure.

Complex 2 crystallize in monoclinic system, *P*2(1)/*n* space group. As shown in Fig. 3b, Sm1 is nine-coordination geometry with an O5–N4 donor set containing four oxygen atoms and four nitrogen atoms from two bpda^2−^ ligands and another oxygen atom from one water molecule, resulting in a distorted tri-capped trigonal prismatic coordination sphere around Sm1 ion. The Sm–O bond lengths are in the range of 2.357(3)–2.659(3) Å and Sm–N ones in 2.545–2.659 Å. The bond angles around Sm ion vary from 62.09(9) to 157.96(9) (see Table S1, ESI† for details). Adjacent samarium ions are bridged through the carboxyl group in the ligand to form one-dimensional chain structure (Fig. 3d).

### 3.3 Thermal gravimetric analysis

**Fig. 4:**
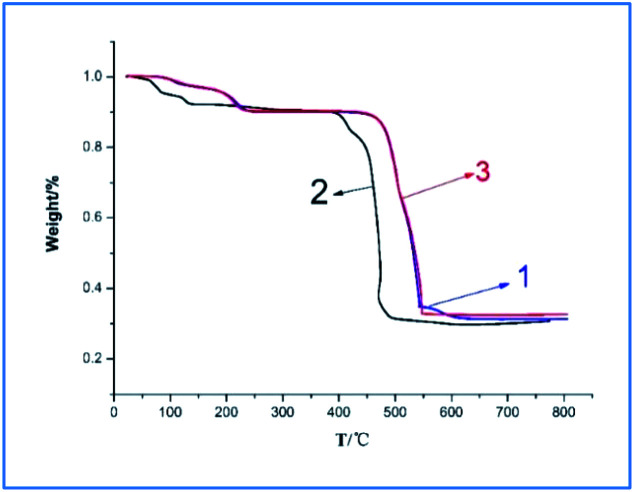
The TGA curves of 1–3.

Fig. 4 is the thermogravimetric analysis diagram of complexes 1–3. Dy-1 and Tb-3 are heterogeneous isomorphism and their TGA curves similar, and then take Dy-1 as an example for analysis. As shown in Fig. 4, Dy-1 starts the first decompose at below 242 °C. The observed weight loss of 9.91% is consistent with the calculated value of 9.7%, which can assign to the decomposition of coordinated water molecules and lattice water molecule. The main mass loss occurs in the temperature range of 460–544 °C with the loss of 54.41%, corresponding to the decomposition of residual organic components of the compound (calculated value, 54.92%), which is consistent with the crystal structure analysis results. The mass percentage of the residue is 34.91%, which is basically consistent with the theoretical calculation value of 34.83% of the oxide, so the final residue is considered as Dy_2_O_3_. For Sm-2, the TG curve exhibits an initial mass loss of 4.74% over the temperature range 55–135 °C, corresponding to the department of the lattice water molecule and coordinated water molecule (calculated. 4.10%). The second main mass loss (58.43%) occurs in 395–491 °C corresponding to the decomposition of the residual organic components of the compound (calculated, 58.5%). The residue is thought to be Sm_2_O_3_ (found 31.05%; calculated 31.43%).

### 3.4 Photoluminescence properties

**Fig. 5:**
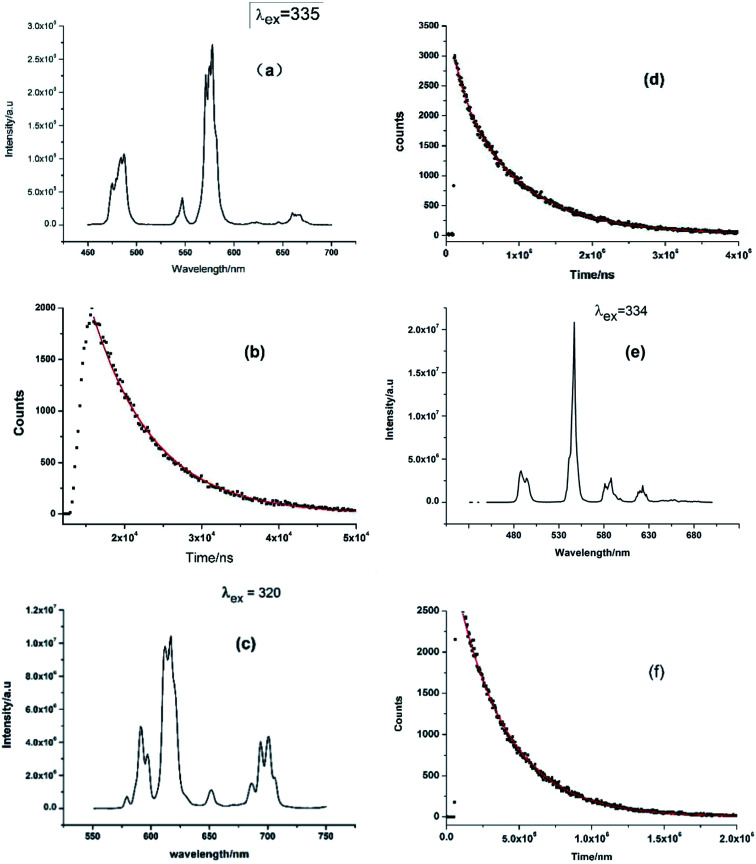
(a) The luminescence emission spectrum of Dy-1. (b) The lifetime decay curve of Dy-1 obtained by monitoring the emission at 577 nm. (c) The luminescence emission spectrum of Sm-2. (d) The lifetime decay curve of Sm-2 obtained by monitoring the emission at 616 nm. (e) The emission spectrum of Tb-3. (f) The lifetime decay curve of Tb-3 obtained by monitoring the emission at 547 nm.

The solid-state emission spectra of the compounds 1–3 were measured at ambient temperature and shown in Fig. 5. As shown in Fig. 5a, the visible region emission from Dy-1 consists of four transitions, ^4^F_9/2_ → ^6^H_15/2_ (magnetic-dipole), ^4^F_9/2_ → ^6^H_13/2_, ^4^F_9/2_ → ^6^H_11/2_ (hypersensitive, electric-dipole) and ^4^F_9/2_ → ^6^H_9/2_, corresponding emission peaks are 487, 546, 577 and 662 nm, respectively. The emission band at 577 nm (^4^F_9/2_ → ^6^H_11/2_) is the strongest among the four bands, which is strongly influenced by the local environment prevailing around Dy(iii) ions.^25^ The emission intensity ratio of ^4^F_9/2_ → ^6^H_11/2_*vs.*^4^F_9/2_ → ^6^H_15/2_ is 2.6 and so high value indicates that this complex lacks a centre of symmetry,^26,27^ since ^4^F_9/2_ → ^6^H_11/2_ is probed to determine coordination symmetry around Dy(iii) system.^28^ To Sm-2, the sharp peaks at 590, 616, 652 and 700 nm should be ascribed to the Sm(iii) transitions of ^4^G_2/5_ → ^6^H_*J*_ (*J* = 5/2, 7/2, 9/2 and 11/2, respectively). The emission band at 616 nm (^4^G_5/2_ → ^6^H_7/2_) is the strongest among the four bands (Fig. 5c). The luminescent emission spectrum of Tb-3 (Fig. 5e) shows four typical bands at about 488, 547, 588 and 623 nm, which correspond to the transitions of the excited state ^5^D_4_ to the ground states ^7^F_*J*_ (*J* = 3, 4, 5, and 6) of the Tb(iii) ions, respectively.^29,30^ Among the four bands, the emission band at 547 nm is the strongest, emitting green light visible to the naked eye under laser lamp.

Fig. 5b, d and f are corresponding luminescence decay curves of complexes 1–3 and they are measured in the condition of the strongest emission peak. The corresponding fluorescence lifetime value (*τ*) obtained by fitting curves of the decay curves on FLS1000 Photoluminescence Spectrometer are listed in Table 2. As shown in Table 2, the experimentally fitted value of *τ* for 2 is 847 μs at 616 nm and it has the longest fluorescence lifetime among the three complexes. It is also a rather better value with comparison to some previous Sm-complex.^31,32^

**Table 2:** Luminescent lifetimes and *χ*^2^ values of complexes 1–3

Complex	*τ* (μs)	*χ* ^2^
Dy-1	8.04	1.099
Sm-2	847.34	1.222
Tb-3	351.31	1.216

The data of solid quantum efficiency measured in the condition of maximum emission for 1–3 are reported in Table 3. The quantum yield of the samarium compound is calculated to be 21.4%, which is much higher than those of the samarium complexes reported in the literature (typically in the range of 1–20%),^13,14,31^ though a small amount of water molecules are involved in the coordination sphere.

**Table 3:** Quantum efficiency and maximum emission of the compounds

Complex	Quantum yield (%)	Maximum emission
Dy-1	1.24	575
Sm-2	21.4	616
Tb-3	15.9	547

### 3.5 Magnetic properties

**Fig. 6:**
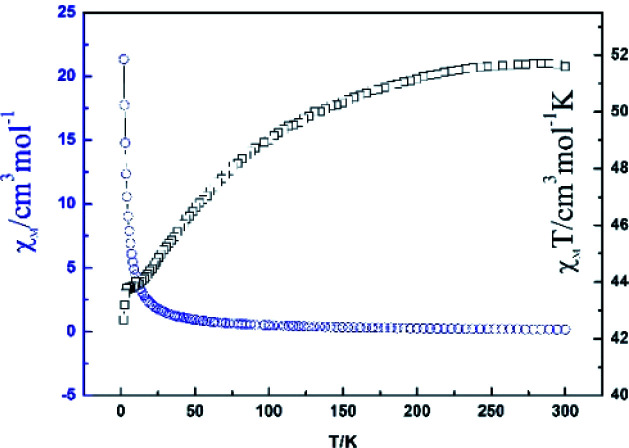
Temperature dependence of the *χ*_MT_ and *χ*_M_ for Dy-1.

The direct current (dc) magnetic susceptibility of Dy-1 was studied in an applied magnetic field of 2000 Oe and the temperature range 300–2 K and plotted as *χ*_MT_*vs. T* in Fig. 6. For Dy-1, the observed *χ*_MT_ value is 51.55 cm^3^ K mol^−1^ at 300 K, which is lower than the expected value of 56.68 cm^3^ K mol^−1^ for four uncoupled Dy(iii) ions (*S* = 5/2, *L* = 5, ^6^H_15/2_, *g* = 4/3). Upon cooling, *χ*_MT_ gradually decreases until 12 K and then drops rapidly to reach a minimum of 42.67 cm^3^ K mol^−1^ at 2 K. The susceptibility *χ*_M_ (blue line in Fig. 6) increases slowly with decreasing temperature and then dramatically increases to 21.37 cm^3^ mol^−1^ at 2 K. This behavior is attributed to antiferromagnetic exchange interaction between the Dy(iii) ions and Stark energy level degeneracy caused by spin–orbit coupling.^33,34^

**Fig. 7:**
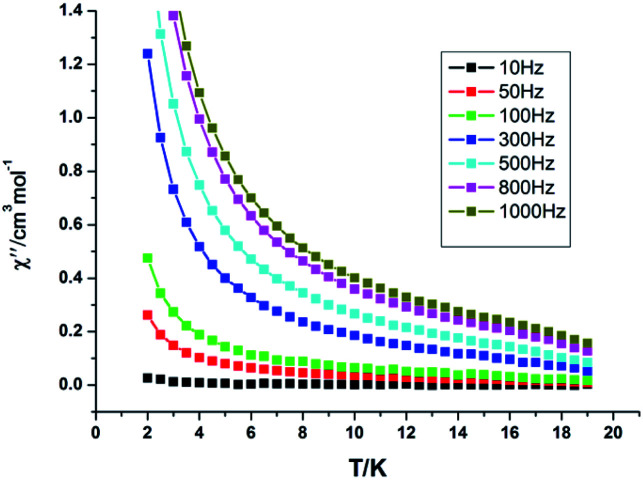
Temperature dependence of out-of-phase (*χ*′′) ac susceptibilities for Dy-1 under a zero dc field.

**Fig. 8:**
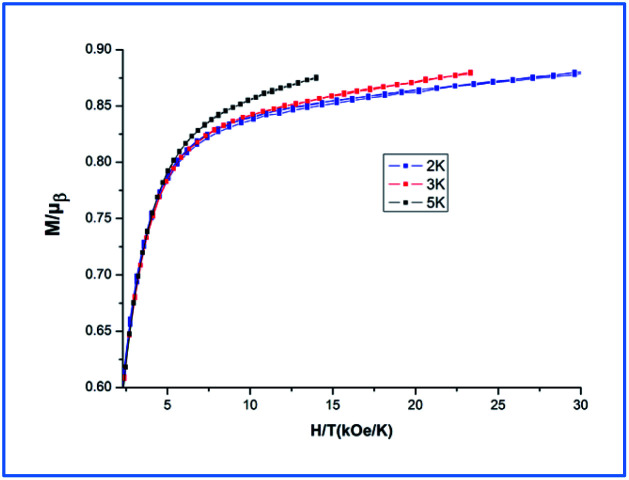
The plot of magnetization *versus* magnetic field of Dy-1 at 2, 3 and 5 K.

The variation of alternating current (ac) susceptibility with frequency and temperature for Dy-1 under 2.2 Oe ac oscillating field was studied, and the magnetization kinetics was investigated (Fig. 7 and 8). The results show that there is magnetic relaxation in complex Dy-1, which is the typical characteristic associated with single molecular magnet behaviour.

The ac susceptibility experiments were carried out in the range 2–19 K and the frequencies selected were 10, 50, 100, 500, 800 and 1000 Hz, respectively. As displayed in Fig. 7 and S1, ESI,† the relaxation time at different temperatures was obtained by fitting the *χ*′′ or *χ*′ *vs.* frequency curves. As shown in Fig. 7, below 8 K, the values of *χ*′ and *χ*′′ keep increasing on cooling, which indicates that there is a slow relaxation of magnetization phenomenon in Dy-1 expected for a single-molecule magnet. Furthermore, the phenomenon of magnetic relaxation becomes more obvious with the increase of magnetic field intensity and the decrease of temperature. However, slow relaxation of magnetization for Dy-1 is observed experimentally only in a narrow temperature range, and no maximum of *χ*′′ is observed in the temperature window, in which the energy barrier and corresponding relaxation time could not be calculated. Alternatively, a method employed by G. Y. Yang *et al.*^35^ can be used to evaluate roughly the energy barrier *E*_a_ and relaxation time *τ*_0_ based on the following relationship (eqn (1)):

1 ln(*χ*′′/*χ*′) = ln(*wτ*_0_) + *E*_a_/*k*_B_*T*

By nonlinear fitting the experimental ln(*χ*′′/*χ*′) *vs.* 1/*T* at different frequencies, we obtained an estimate of the activation energy *E*_a_/*k*_B_ = 1.14 K and *τ*_0_ = 1.2 × 10^−6^ s. A more precise result must wait for very low temperature measurements (*T* < 1 K) by using a micro-SQUID.

As described in 3.2 structural descriptions section, Dy1 is coordinated to four nitrogen atoms and four oxygen atoms while Dy2 is connected to two nitrogen atoms and six oxygen atoms. Due to the bond lengths are different after Dy(iii) coordinating to nitrogen or oxygen atoms, the symmetry and intensity of Dy(iii) coordination field are different, which may strongly impact on magnetic anisotropy, leading to distinct dynamic behavior.^36,37^ It is visible that magnetic relaxation mainly results from Dy(iii) anisotropy that is very sensitive to changes of the coordination geometry.

Additionally, magnetization data (*M*) for Dy-1 were collected in the field range 0–70 kOe and at 2, 3 and 5 K, as shown in the Fig. 8, the magnetization measurements of Dy-1 increase rapidly for low fields then increases gently to a value of 20.89 Nβ for Dy-1 at 2 K and 70 kOe. This value is lower than the expected saturation value of 40 μB, but close to four uncorrelated Dy ions' magnetic moments (4 × 5.23 μB), which is likely due to crystal-field effects and the low-lying excited states.^38^ At 2 K, the *M versus H* data of Dy-1 exhibit slim butterfly-shaped hysteresis loops without a remanence and a coercive field (Fig. S2, ESI†). This lack is due to the slow sweep rate of the loop compared with the fast zero-field relaxation.^39,40^ The non-superposition of the *M versus HT*^−1^ curves obtained (Fig. 8) suggests the significant magneto-anisotropy and a low-lying excited state present in Dy-1.^41–43^

## 4. Conclusion

This article reports the syntheses, crystal structures, fluorescence and magnetic properties of three Ln(iii) compounds base on H_2_bpda ligand. Complexes 1, 2 and 3 exhibit strong f–f transition and long luminescent lifetime and high quantum efficiency, which indicated that the ligand H_2_bpda was a good organic chelator to absorb and transfer energy to Dy(iii), Sm(iii), Tb(iii) and could be considered as promising candidate in the design of photoluminescence devices. Dy-1 exhibit slow magnetization relaxation and single molecular magnet behaviour except showing obvious yellow emission, which it may be a candidate of photo-magnetic functional.

## Conflicts of interest

There are no conflicts to declare.

## Supplementary Material

RA-010-C9RA10975G-s001

RA-010-C9RA10975G-s002

## References

[cit1] Liu Y., Tu D., Zhu H., Chen X. (2013). Lanthanide-doped luminescent nanoprobes: controlled synthesis, optical spectroscopy, and bioapplications. Chem. Soc. Rev..

[cit2] Feng X., Guo N., Chen H. P., Wang H., Yue L., Chen X., Ng S. W., Liu X., Ma L. F., Wang L. Y. (2017). A series of anionic host coordination polymers based on azoxybenzene carboxylate: structures, luminescence and magnetic properties. Dalton Trans..

[cit3] Liu X. F., Du L. Y., Wang Y. F., Li R. F., Feng X., Ding Y. Q. (2019). J. Mol. Struct..

[cit4] Cui Y., Chen B., Qian G. (2014). Lanthanide metal-organic frameworks for luminescent sensing and light-emitting applications. Coord. Chem. Rev..

[cit5] Schaferling M. (2012). The Art of Fluorescence Imaging with Chemical Sensors. Angew. Chem., Int. Ed..

[cit6] Wang X., Chang H., Xie J., Zhao B., Liu B., Xu S., Pei W., Ren N., Huang L., Huang W. (2014). Recent developments in lanthanide-based luminescent probes. Coord. Chem. Rev..

[cit7] Feng X., Feng Y., Guo N., Sun Y. L., Zhang T., Ma L. F., Wang L. Y. (2017). Series d–f heteronuclear metal-organic frameworks: color tunability and luminescent probe with switchable properties. Inorg. Chem..

[cit8] Zinna F., Giovanella U., Bari L. D. (2015). Highly Circularly Polarized Electroluminescence from a Chiral Europium Complex. Adv. Mater..

[cit9] Xu H., Wang J. Z., Wei Y., Xie G. H., Xue Q., Deng Z. P., Huang W. (2015). A unique white electroluminescent one-dimensional europium(III) coordination polymer. J. Mater. Chem. C.

[cit10] Feng X., Li R. F., Wang L. Y. (2015). A series of homonuclear lanthanide coordination polymers based on a fluorescent conjugated ligand: syntheses, luminescence and sensor. CrystEngComm.

[cit11] Coban M. B., Amjad A., Aygun M., Kara H. (2017). Sensitization of Ho^III^ and Sm^III^ luminescence by efficient energy transfer from antenna ligands: magnetic, visible and NIR photoluminescence properties of Gd^III^, Ho^III^ and Sm^III^ coordination polymers. Inorg. Chim. Acta.

[cit12] Räsänen M., Takalo H., Rosenberg J., Mäkelä J., Haapakka K., Kankare J. (2014). Study on photophysical properties of Eu(III) complexes with aromatic β-diketones-role of charge transfer states in the energy migration. J. Lumin..

[cit13] Wang A. L., Zhou D., Chen Y. N., Li J. J., Zhang H. X., Zhao Y. L., Chu H. B. (2016). Crystal structure and photoluminescence of europium, terbium and samarium compounds with halogen-benzoate and 2,4,6-tri(2-pyridyl)-s-triazine. J. Lumin..

[cit14] Hou K. L., Bai F. Y., Xing Y. H., Wang J. L., Shi Z. (2011). A novel family of 3D photoluminescent lanthanide-bta-flexible MOFs constructed from 1,2,4,5-benzenetetracarboxylic acid and different spanning of dicarboxylate acid ligands. CrystEngComm.

[cit15] Li X. L., Wu J. F., Tang J. K., Boris L. G., Shi W., Cheng P. (2016). A planar triangular Dy3 + Dy3 single-molecule magnet with a toroidal magnetic moment. Chem. Commun..

[cit16] Lin P. H., Sun W. B., Tian Y. M., Yan P. F., Ungur L., Chibotaru L. F., Murugesu M. (2012). Ytterbium can relax slowly too: a field-induced Yb2 single-molecule magnet. Dalton Trans..

[cit17] Meng Y., Chen Y. C., Zhang Z. M., Lin Z. J., Tong M. L. (2014). Gadolinium Oxalate Derivatives with Enhanced Magnetocaloric Effect *via* Ionothermal Synthesis. Inorg. Chem..

[cit18] He J. Q., Xie S. F., Lai B. L., Yang M., Chen W. B., Zhang Y. Q., Dong W. (2018). A new salicylaldehyde-based azo dye and its two lanthanide(III) complexes displaying slow magnetic relaxation. Dalton Trans..

[cit19] Sheldrick G. M. , SHELXL-2014/7, Program for the Solution of Crystal Structure, University of Göttingen, Germany

[cit20] Li R. F., Zhu X. X., Liu X. F., Feng X., Wang L. Y. (2019). Synthesis, Crystal Structure and Fluorescence Properties of a Terbium(III) Complex with Biphenyl-2,3,3′,5′-tetracarboxylic Acid. Chin. J. Struct. Chem..

[cit21] Lu Y. W. and Deng Z. H., Practical Infrared Spectrum Parse, Publishing House of Electronics Industry, Beijing, 1989

[cit22] Teoh S. G., Ang S. H., Declercq J. P. (1997). Synthesis and characterization of di-*n*-butylbis(2,4-dihydroxybenzoato tin(iv). Polyhedron.

[cit23] Yue B., Chen Y. N., Chu H. B., Qu Y. R., Wang A. L., Zhao Y. L. (2014). Synthesis, crystal structures and fluorescence properties of dinuclear Tb(III) and Sm(III) complexes with 2,4,6-tri(2-pyridyl)-1,3,5-triazine and halogenated benzoic acid. Inorg. Chim. Acta.

[cit24] Wang A. L., Wei X. Y., Zhang H. X., Yue B., Qu Y. R., Kang J., Wang Z. X., Chu H. B., Zhao Y. L. (2014). Crystal structure and photoluminescence of two europium compounds with phenoxyacetic acid and 2,4,6-tri(2-pyridyl)-s-triazin. Dalton Trans..

[cit25] Bag P., Rastogi C. K., Biswas S., Sivakumar S., Mereacre V., Chandrasekhar V. (2015). Homodinuclear Lanthanide {Ln2} (Ln = Gd, Tb, Dy, Eu) Complexes Prepared from a *o*-Vanillin based Ligand: Luminescence and Single-Molecule Magnetism Behavior. Dalton Trans.

[cit26] Ahmed Z., Iftikhar K. (2015). Efficient Layers of Emitting Ternary Lanthanide Complexes for Fabricating Red, Green, and Yellow OLEDs. Inorg. Chem..

[cit27] Raju G. S. R., Park J. Y., Jung H. C., Yang H. K., Moon B. K., Jeong J. H., Kim J. H. (2009). Synthesis and luminescent properties of low concentration Dy3+:GAP nanophosphors. Opt. Mater..

[cit28] Gu F., Wang S. F., Lu M. K., Zhou G. J., Xu D., Yuan D. R. (2004). Structure Evaluation and Highly Enhanced Luminescence of Dy^3+^-Doped ZnO Nanocrystals by Li^+^ Doping *via* Combustion Method. Langmuir.

[cit29] Feng X., Ma L. F., Liu L., Wang L. Y., Song H. L., Xie S. Y. (2013). A Series of Heterometallic Three-Dimensional Frameworks Constructed from Imidazole-Dicarboxylate: Structures, Luminescence, and Magnetic Properties. Cryst. Growth Des..

[cit30] Feng X., Chen J. L., Wang L. Y., Xie S. Y., Yang S., Huo S. Z., Ng S. W. (2014). A series of homonuclear lanthanide complexes incorporating isonicotinic based carboxylate tectonic and oxalate coligand: structures, luminescent and magnetic properties. CrystEngComm.

[cit31] Feng J., Yu J. B., Song S. Y., Sun L. N., Fan W. Q., Guo X. M., Dang S., Zhang H. J. (2009). Near-infrared luminescent xerogel materials covalently bonded with ternary lanthanide [Er(iii), Nd(iii), Yb(iii), Sm(iii)] complexes. Dalton Trans..

[cit32] Chow C. Y., Eliseeva S. V., Trivedi E. R., Nguyen T. N., Kampf J. W., Petoud S., Pecoraro V. L. (2016). A Promising Family of Highly Luminescent Lanthanide Complexes that Covers
Visible and Near-Infrared Domains. J. Am. Chem. Soc..

[cit33] Lin S. Y., Zhao L., Guo Y. N., Zhang P., Guo Y., Tang J. K. (2012). Two New Dy3 Triangles with Trinuclear Circular Helicates and Their Single-Molecule Magnet Behavior. Inorg. Chem..

[cit34] Du C. C., Wang X. F., Zhou S. B., Jia D. Z. W. D. (2017). New complexes constructed from *in situ* nitration of (1H-tetrazol-5-yl)phenol: synthesis, structures and properties. CrystEngComm.

[cit35] Li H. L., Liu Y. J., Liu J. L., Chen L. J., Zhao J. W., Yang G. Y. (2017). Structural Transformation from Dimerization to Tetramerization of Serine-decorated Rare-earth Incorporated Arsenotungstates Induced by the Usage of Rare-earth Salts. Chem.–Eur. J..

[cit36] Chen G. J., Gao C. Y., Tian J. L., Tang J., Gu W., Liu X., Yan S. P., Liao D. Z., Cheng P. (2011). Dalton Trans..

[cit37] Ma Y., Xu G. F., Yang X., Li L. C., Tang J., Yan S. P., Cheng P., Liao D. Z. (2010). Chem. Commun..

[cit38] Liu S. J., Zhao J. P., Song W. C., Han S. D., Liu Z. Y., Bu X. H. (2013). Slow Magnetic Relaxation in Two New 1D/0D DyIII Complexes with a Sterically Hindered Carboxylate Ligand. Inorg. Chem..

[cit39] Bi Y., Guo Y. N., Zhao L., Guo Y., Lin S. Y., Jiang S. D., Tang J., Wang B. W., Gao S. (2011). Chem.–Eur. J..

[cit40] Zhang S., Ke H., Sun L., Li X., Shi Q., Xie G., Wei Q., Yang D., Wang W., Chen S. (2016). Magnetization Dynamics Changes of Dysprosium(III) Single-Ion Magnets Associated with Guest Molecules. Inorg. Chem..

[cit41] Lin S. Y., Guo Y. N., Guo Y., Zhao L., Zhang P., Keand H. S., Tang J. K. (2012). Macrocyclic ligand encapsulating dysprosium triangles: axial ligands perturbed magnetic dynamics. Chem. Commun..

[cit42] Liu J. L., Chen Y. C., Tong M. L. (2018). Symmetry strategies for high performance lanthanide-based single-molecule magnets, a series of heterometallic three-dimensional frameworks constructed from imidazole-dicarboxylate: structures, luminescence, and magnetic properties. Chem. Soc. Rev..

[cit43] He J. Q., Xie S. F., Lai B. L., Yang M., Chen W. B., Zhang Y. Q., Dong W. (2018). A new salicylaldehyde-based azo dye and its two lanthanide(III) complexes displaying slow magnetic relaxation. Dalton Trans..

