# Correction: Lanthanide complexes based on a conjugated pyridine carboxylate ligand: structures, luminescence and magnetic properties

**DOI:** 10.1039/d0ra90017f

**Published:** 2020-02-27

**Authors:** Rong-fang Li, Rui-hao Li, Xin-fang Liu, Xin-hong Chang, Xun Feng

**Affiliations:** College of Chemistry and Chemical Engineering, Henan Key Laboratory of Function Oriented Porous Materials, Luoyang Normal University Luoyang Henan 471934 China fengx@lynu.edu.cn

## Abstract

Correction for ‘Lanthanide complexes based on a conjugated pyridine carboxylate ligand: structures, luminescence and magnetic properties’ by Rong-fang Li *et al.*, *RSC Adv.*, 2020, **10**, 6192–6199.

The authors regret that there were some errors in the references in the original article. On page 6197, the text “Alternatively, a method employed by G. Y. Yang *et al.*^35^” should read “Alternatively, a method employed by J. K. Tang *et al.*^35^”. The citation to ref. 41–43 before the conclusions should be changed to ref. 41 and 42.

In the references section, the original ref. 35 should be deleted, and the original ref. 41 should be renumbered as ref. 35. The original ref. 42 and 43 should also be renumbered as ref. 41 and 42, respectively.

The Royal Society of Chemistry apologises for these errors and any consequent inconvenience to authors and readers.

## Supplementary Material

