# Supplementary material for: Lanthanide complexes based on a conjugated pyridine carboxylate ligand: structures, luminescence and magnetic properties
Source: RSC Adv. 2020 Feb 10;10(11):6192–9. doi: 10.1039/c9ra10975g (PMC9049638; doi:10.1039/c9ra10975g)
Supplement: RA-010-C9RA10975G-s001 [file RA-010-C9RA10975G-s001.pdf]

Electronic Supplementary Material (ESI) for RSC Advances.
This journal is © The Royal Society of Chemistry 2020

Supporting Information for

Lanthanide complexes based on conjugated pyridine carboxylate ligand: structures, luminescence and magnetic properties

Rong-fang Li, Rui-hao Li, Xin-fang Liu, Xin-hong Chang, Xun Feng

College of Chemistry and Chemical Engineering and Henan Key Laboratory of Function Oriented Porous Materials, Luoyang Normal University, Luoyang, 471022, *China*

1. Additional Table

Table S1 Selected bond lengths [Å] and bond angles [º] for compounds **1- 3**.

		**1**			
Dy1-O3	2.295(4)	Dy3-O18	2.277(4)	Dy4-N10	2.489(5)
Dy2-O16	2.390(4)	Dy1-O6	2.316(4)	Dy2-N6	2.487(4)
Dy1-O8	2.354(4)	Dy3-O21	2.288(4)	Dy4-N12	2.481(5)
Dy3-O20	2.365(4)	Dy3-O22	2.410(4)	Dy2-N5	2.488(4)
Dy2-O15	2.375(4)	Dy3-O23	2.388(4)	Dy4-N11	2.501(4)
Dy1-N3	2.493(4)	Dy1-O4	2.291(2)	Dy3-N7	2.479(4)
Dy2-O9	2.379(4)	Dy4-O25	2.383(4)	Dy1-N1	2.457(5)
Dy2-O10	2.299(4)	Dy4-O26	2.291(4)	Dy1-N2	2.489(5)
Dy2-O12	2.322(4)	Dy4-O28	2.325(4)	Dy4-N9	2.464(4)
Dy4-O30	2.304(4)	Dy2-O14	2.384(4)	Dy1-N4	2.461(5)
Dy3-N8	2.487(4)	Dy3-O19	2.365(4)	O18-Dy3-O22	83.97(15)
O3-Dy1-O4	88.35(17)	O3-Dy1-N1	79.45(16)	O18-Dy3-O19	86.78(14)
O3-Dy1-O6	92.46(17)	O3-Dy1-N2	82.85(16)	O18-Dy3-O20	83.40(15)
O3-Dy1-O8	163.30(14)	O3-Dy1-N3	131.04(16)	O18-Dy3-O21	163.17(14)
O4-Dy1-N3	80.67(16)	O4-Dy1-N4	79.43(17)	O19-Dy3-N8	78.57(14)
O4-Dy1-N2	131.06(16)	O19-Dy3-N7	75.78(14)	O19-Dy3-O23	146.28(14)
O4-Dy1-O8	92.45(17)	O19-Dy3-O22	141.02(13)	O4-Dy1-N1	67.45(18)
O4-Dy1-O6	163.24(17)	O19-Dy3-O20	71.08(15)	O18-Dy3-N7	66.45(14)
O3-Dy1-N4	67.53(15)	O18-Dy3-N8	129.88(15)	O18-Dy3-O23	92.81(14)
O6-Dy1-O8	91.55(16)	O6-Dy1-N1	129.13(17)	O20-Dy3-O22	70.27(15)
O20-Dy3-O23	142.40(15)	O20-Dy3-N7	136.02(15)	O12-Dy2-N6	129.36(14)
O6-Dy-1N2	65.58(15)	O20-Dy3-N8	133.36(16)	O14-Dy2-O16	142.00(14)
O6-Dy1-N3	86.22(15)	O21-Dy3-O19	92.23(15)	O14-Dy2-N5	73.74(14)
O6-Dy1-N4	85.41(16)	O21-Dy3-O20	80.39(16)	O14-Dy2-N6	73.47(14)
O8-Dy1-N1	85.46(15)	O21-Dy3-O22	86.31(15)	O15-Dy2-O9	74.51(14)

O8-Dy1-N2	84.07(15)	O21-Dy3-O23	97.24(14)	O15-Dy2-O14	145.68(15)
O8-Dy1-N3	65.41(15)	O21-Dy3-N7	129.51(14)	O15-Dy2-O16	71.77(15)
O8-Dy1-N4	128.99(15)	O21-Dy3-N8	66.05(15)	O15-Dy2-N5	126.82(15)
N1-Dy1-N2	63.60(17)	O22-Dy3-N7	132.59(15)	O15-Dy2-N6	138.05(14)
N1-Dy1-N3	135.74(17)	O22-Dy3-N8	134.26(14)	O16-Dy2-N5	74.36(14)
N1-Dy1-N4	133.51(17)	O23-Dy3-O22	72.12(13)	O16-Dy2-N6	74.26(14)
N2-Dy1-N3	138.02(15)	O23-Dy3-N7	73.24(14)	N6-Dy2-N5	63.84(14)
N4-Dy1-N2	137.61(16)	O23-Dy3-N8	75.98(14)	O28-Dy4-O25	87.90(15)
N4-Dy1-N3	63.57(16)	N7-Dy3-N8	63.52(15)	O28-Dy4-N9	129.24(15)
O9-Dy2-O14	72.56(14)	O25-Dy4-N9	83.93(14)	O28-Dy4-N10	65.98(14)
O9-Dy2-O16	145.32(14)	O25-Dy4-N10	80.91(14)	O28-Dy4-N11	83.42(14)
O9-Dy2-N5	134.99(14)	O25-Dy4-N11	65.32(14)	O28-Dy4-N12	85.46(15)
O9-Dy2-N6	130.46(15)	O25-Dy4-N12	129.06(13)	O12-Dy2-O9	87.93(15)
O10-Dy2-O9	81.84(14)	O26-Dy4-O25	90.54(16)	O12-Dy2-O14	93.87(14)
O10-Dy2-O12	164.48(14)	O26-Dy4-O28	163.88(14)	O12-Dy2-O15	75.47(14)
O10-Dy2-O14	94.15(14)	O26-Dy4-O30	90.35(16)	O12-Dy2-O16	91.28(14)
O10-Dy2-O15	90.50(14)	O26-Dy4-N9	66.43(15)	O12-Dy2-N5	65.54(14)
O10-Dy2-O16	90.56(14)	O26-Dy4-N10	129.55(14)	O10-Dy2-N6	65.85(14)
O10-Dy2-N5	129.64(15)	O26-Dy4-N11	81.35(15)	O26-Dy4-N12	83.08(15)

2

Sm1-N1	2.549(3)	Sm1 -O8	2.381(2)	Sm1-O6	2.659(3)
Sm1-N2	2.545(3)	Sm1 -O4	2.357(3)	Sm1-O3	2.451(2)
Sm1-N3	2.614(3)	Sm1- O2^{1}	2.438(3)	Sm1-N4	2.659(3)
N2-Sm1-N1	62.09(9)	N3-Sm1-N1	114.70(9)	N3-Sm1-N2	131.55(9)
N4-Sm1-N1	144.31(9)	N4-Sm1-N2	150.56(9)	N4-Sm1-N3	59.93(9)
O2^{1}-Sm1-N1	69.51(9)	O2^{1}-Sm1-N2	93.50(9)	O2^{1}-Sm1-N3	132.63(9)
O2^{1}-Sm1-N4	88.95(9)	O3-Sm1-N1	74.27(8)	O3-Sm1-N2	70.80(8)
O3-Sm1-N3	62.86(8)	O3 -Sm1- N4	120.96(8)	O3-Sm1-O2^{1}	143.73(9)
O4-Sm1-N1	125.67(9)	O6-Sm1-N2	94.38(9)	O8-Sm1-O6	124.70(9)
O4-Sm1-N2	64.97(9)	O6-Sm1-N3	115.83(9)	O8-Sm1-O4	157.96(9)
O4-Sm1-N3	91.50(10)	O6-Sm1-N4	60.23(9)	O8-Sm1-O3	87.79(8)
O4-Sm1-N4	89.97(9)	O6-Sm1-O2^{1}	65.23(9)	O8-Sm1-O2^{1}	75.17(9)
O4-Sm1-O2^{1}	125.73(10)	O6-Sm1-O3	145.53(9)	O8-Sm1-N4	82.97(9)
O4-Sm1-O3	77.99(9)	O6-Sm1-O4	67.55(10)	O8-Sm1-N3	66.88(8)
O6-Sm1-N1	127.00(9)	O8-Sm1-N1	64.54(8)	O8-Sm1-N2	125.99(9)

3

Tb1-O2	2.304(2)	Tb1-N2	2.507(3)	Tb2-O9	2.392(2)
Tb3-O17	2.320(2)	Tb3-N7	2.491(3)	Tb4-O24	2.425(2)
Tb1-O4	2.331(3)	Tb1-N3	2.505(3)	Tb2-O10	2.403(2)
Tb3-O19	2.331(2)	Tb3-N8	2.511(2)	Tb4-O25	2.400(2)
Tb1-O6	2.315(3)	Tb1-N4	2.480(3)	Tb2-O11	2.336(2)

Tb3-O21	2.313(3)	Tb3-N9	2.515(3)	Tb4-O26	2.294(2)
Tb1-O8	2.375(2)	Tb1-N1	2.4609(15)	Tb2-O13	2.410(2)
Tb3-O23	2.402(2)	Tb3-N10	2.488(3)	Tb4-O28	2.307(2)
Tb2-O14	2.396(2)	Tb4-O30	2.378(2)	Tb2-O15	2.315(2)
Tb4-O31	2.388(2)	Tb2-N5	2.507(2)	Tb4-N11	2.495(3)
Tb2- N6	2.497(3)	Tb4-N12	2.503(3)		
O23-Tb3-N8	65.25(8)	O8-Tb1-N4	85.63(9)	O23-Tb3- N9	80.52(8)
O8-Tb1-N2	65.31(8)	O23-Tb3-N7	128.53(8)	O8-Tb1-N3	84.10(9)
O21-Tb3-N9	129.21(8)	O6-Tb1-N1	79.89(9)	O21-Tb3-N10	66.31(9)
O6-Tb1-N3	130.00(9)	O21-Tb3-N8	81.55(9)	O6-Tb1-N4	66.71(10)
O21-Tb3-O23	90.42(9)	O6-Tb1-N2	81.24(9)	O21-Tb3-N7	83.16(9)
O17-Tb3-N8	129.31(8)	O6-Tb1-O4	164.37(9)	O21-Tb3-O19	164.46(8)
O4-Tb1-N1	85.94(8)	O21-Tb3-O17	90.22(9)	O6-Tb1-O8	92.18(10)
O2-Tb1-N2	130.05(9)	O4-Tb1-N4	128.75(10)	O19-Tb3-N10	128.61(9)
O17-Tb3-N7	66.09(8)	O4-Tb1-N3	65.51(9)	O19-Tb3-N9	65.51(8)
O2-Tb1-O8	164.34(8)	O4-Tb1-N2	86.54(9)	O19-Tb3-N8	83.55(8)
O17-Tb3-O23	165.31(7)	O4-Tb1-O8	91.52(9)	O19-Tb3-N7	86.22(9)
O2-Tb1-O6	87.98(10)	O2-Tb1-N1	66.77(8)	O19-Tb3-O23	87.34(9)
O17-Tb3-O19	95.82(9)	O2-Tb1-N4	80.05(9)	O17-Tb3-N10	82.69(9)
O2-Tb1-O4	92.51(10)	O2-Tb1-N3	83.82(9)	O17-Tb3-N9	87.72(9)
O23-Tb3-N10	84.14(8)	N3-Tb1-N2	138.16(9)	N7-Tb3-N8	63.29(9)
N4-Tb1-N2	135.81(10)	N7-Tb3-N9	139.59(9)	N4-Tb1-N3	63.29(10)
N8-Tb3-N9	134.80(9)	N1-Tb1-N2	63.35(8)	N10-Tb3-N7	136.17(9)
N1-Tb1-N3	138.15(9)	N10-Tb3-N8	135.37(9)	N1-Tb1-N4	133.41(9)
N10-Tb3-N9	63.10(9)	O9-Tb2-O10	72.72(8)	O24-Tb4-N11	132.93(9)
O9-Tb2-O13	145.69(8)	O24-Tb4-N12	134.49(8)	O9-Tb2-O14	73.72(9)
O25-Tb4-O24	72.08(8)	O9-Tb2-N5	135.22(8)	O25-Tb4-N11	73.69(9)
O9-Tb2-N6	130.80(9)	O25-Tb4-N12	75.99(8)	O10-Tb2-O13	141.41(8)
O26-Tb4-O24	84.42(9)	O10-Tb2-N5	74.19(8)	O26-Tb4-O25	92.98(9)
O11-Tb2-O14	76.20(8)	O26-Tb4-N12	129.18(9)	O28-Tb4-O24	86.63(9)
O26-Tb4-N11	66.11(9)	O11-Tb2-N5	65.28(8)	O11-Tb2-N6	128.66(8)
O11-Tb2-O13	90.87(9)	O28-Tb4-O25	97.06(9)	O13-Tb2-N5	73.86(8)
O26-Tb4-O31	86.35(9)	O28-Tb4-O30	81.75(9)	O13-Tb2-N6	73.69(9)
O11-Tb2-O10	94.97(8)	O28-Tb4-O31	92.04(9)	O14-Tb2-O10	145.48(8)
O26-Tb4-O30	82.51(9)	O28-Tb4-N11	129.03(9)	O14-Tb2-O13	72.82(9)
O11-Tb2-O9	88.46(9)	O28-Tb4-N12	65.96(9)	O14-Tb2-N5	127.76(9)
O10--Tb2-N6	72.98(8)	O26-Tb4-O28	163.82(8)	O30-Tb4-O24	69.84(9)
O14-Tb2-N6	138.11(9)	O30-Tb4-O25	141.91(9)	O15-Tb2-O9	82.04(8)
O30-Tb4-O31	70.82(9)	O15- Tb2-O10	92.86(9)	O30-Tb4-N11	135.25(9)
O15-Tb2-O11	165.33(8)	O30-Tb4-N12	134.55(10)	O15-Tb2-O13	90.72(9)
O31-Tb4-O24	140.41(8)	O15-Tb2-O14	90.36(8)	O31-Tb4-O25	146.94(8)

2. Additional figures

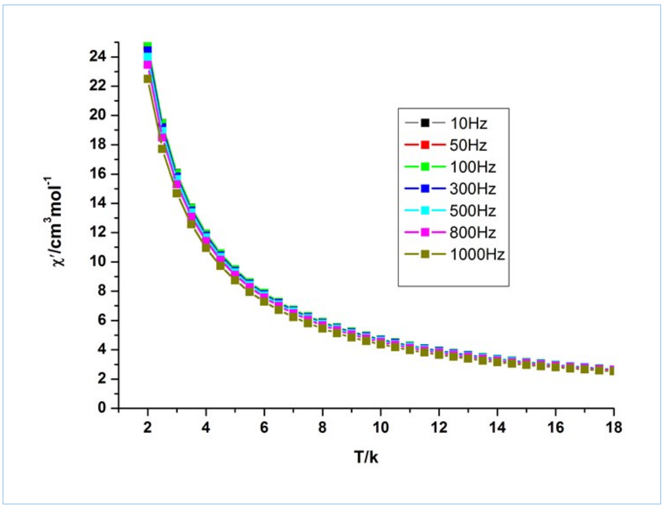

Figure S1 Frequency dependence of in-phase components of ac susceptibility at different temperature for Dy-**1** under zero dc field.

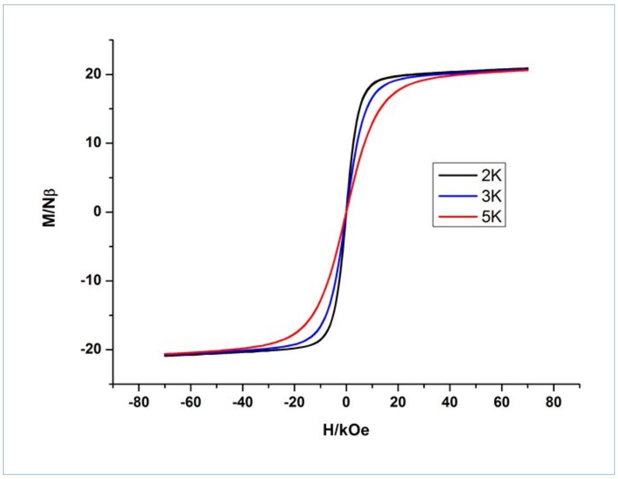

Figure S2 View of M versus HT^{-1} plots for compound Dy-**1.**
